# Improvements in lower-limb muscle strength and foot pressure distribution with foot care in frail elderly adults: a randomized controlled trial from Japan

**DOI:** 10.1186/s12877-019-1097-z

**Published:** 2019-03-14

**Authors:** Tomoko Yamashita, Kazuhiko Yamashita, Chugo Rinoie, Yoshimasa Takase, Mitsuru Sato, Kenji Yamada, Yoshiki Sawa

**Affiliations:** 1 Graduate School of Medicine, Osaka University, 1-3 Yamadaoka, Suita, Osaka 565-0871 Japan; 2 Department of Podiatric Surgery, Methodist Hospital of Southern California, 300 W Huntington Drive, Arcadia, California 91007 USA; 3 Takase Clinic, 1-16-6 Shimomaruko, Ota-ku, Tokyo, 146-0092 Japan; 4 School of Nursing and Rehabilitation Sciences, Showa University, 1865 Tookaichibacho, Midori, Yokohama, Kanagawa 226-8555 Japan

**Keywords:** Frail elderly adult, Foot care, Foot pressure distribution, Lower limbs, Muscle strength

## Abstract

**Background:**

Abnormalities in the feet and toenails are common among the elderly and may increase the risk of falls. This study aimed to investigate the changes in toe-gap force, knee-gap force, foot pressure distribution, the ability to perform activities of daily living, subjects’ feelings and behaviors, and physical function resulting from daily lifestyle modification and foot care.

**Methods:**

The study participants included 74 elderly adults (mean age 80.3 ± 7.5 years) with foot problems who had been divided into three groups based on Japan’s nursing care insurance system levels: certified ineligible for support, eligible for support, or eligible for long-term care. Additionally, a control group of 106 elderly adults in good health was recruited. The differences between the intervention and control groups was examined using the Student’s t-test, and differences between the three intervention subgroups and the control group were examined using one-way analysis of variance.

**Results:**

After intervention, abnormalities in the participants’ feet and toenails improved. Significant increases in lower-limb muscle strength were observed, and foot pressure distribution had improved. The foot-care intervention significantly improved lower-limb muscle strength and decreased the risk of falling, even in elderly adults whose physical function had deteriorated.

**Conclusion:**

In frail elderly adults, care of the feet and toenails can improve lower-limb muscle strength and foot pressure distribution. In addition, the individuals’ social participation increased, and their behavior improved.

**Trial registration:**

University hospital Medical Information Network- Clinical Trials (UMIN-CTR) with the number: UMIN000034742. Registration date: 11/01/2018.

## Background

Studies have suggested that foot disorders currently affect between 71 and 87% of older patients and frequent require medical and foot care [1–4]. The incidence of foot problems is increasing as a consequence of increasing life expectancy. Abnormalities in elderly feet and toenails, including toenail thickening, wounds, and curvature (i.e., pincer toenails), can diminish lower-limb muscle strength and balance, increasing the risk of falls [5, 6]. These issues have a negative influence on functional capacity and quality of life [7–9]. Diseases and disorders of the foot and its related anatomical structures in older patients cause pain and limit the individuals’ function, which can affect their quality of life, dignity, and ability to remain independent [10]. Risk factors for falls include age, sex, and medication. In terms of physical aspects, the risk factors include lower-limb muscle strength, walking ability, and balance [11, 12]. In addition, fractures sustained in falls have been identified as a key factor in reduction in the ability to perform activities of daily living (ADL) and increase in medical expenses in elderly adults. As falls cause 90% of the fractures sustained by elderly adults, the prevention of falls is of the utmost importance.

Reducing the risk of falling via the administration of foot and toenail care is critical to the improvement and maintenance of physical function and ADL in elderly adults. Most previous studies have focused on providing foot care education to diabetic patients; however, few studies have examined the effects on the risk of falling in the frail elderly population that receives foot care [13, 14]. Although studies on the changes in physical function due to foot care have been carried out, none have examined its influence on the patients’ behavior and attitude.

In this study we provided foot care to elderly adults who have problems with their feet and toenails for the purpose of in order to help reduce the risk of falls. We conducted a questionnaire survey to examine changes in fall-related physical function, lower-limb muscle strength, foot pressure distribution, the ability to perform ADL, patients’ feelings and behaviors, and physical function resulting from the treatment. We hypothesized that foot care would reduce the risk of falling in elderly patients with foot conditions that affected balance, increase activity, and improve behaviors.

## Methods

### Subjects

This study was randomized parallel-group trial. In this study, elderly adults were defined as those older than 65 years. All participants in the intervention group were recruited from a senior center. They were able to stand upright and walk unaided. Those who could not stand upright and walk unaided, had severe dementia, could not have their physical function measured, or did not have their physicians’ permission were excluded. The intervention group included 74 elderly adults with foot problems who had been certified ineligible for support, eligible for support, or eligible for long-term care via the nursing care insurance system in Japan. To allow for comparisons, a control group of 106 healthy elderly adults was also recruited from a pool of 1093 participants in a city health project. Control subjects were selected via propensity matching using the following factors: age and sex for certified ineligible for support.

The intervention and control groups’ mean ages were 83.0 ± 7.5 (range, 66–98) years and 77.3 ± 5.7 (range, 65–88) years, respectively, and a significant age difference was found between the two groups (*p* < 0.01). The intervention group included three subgroups containing 32, 13, and 29 subjects who were certified ineligible for support (mean age, 78.8 ± 7.2; range, 66–96 years), eligible for support (mean age, 86.8 ± 3.2; range, 80–92 years), and eligible for long-term care (mean age, 85.8 ± 6.7; range, 73–98 years), respectively. Age did not differ significantly between the control group and the subgroup certified ineligible for care; however, the mean ages of the control group and the subgroups certified eligible for support and long-term care differed significantly (*p* < 0.01).

Classification into “eligible for support” and “eligible for long-term care” is performed for all elderly individuals who participate in the nursing care insurance system in Japan. Although individuals who are eligible for support may not require nursing care, they experience inconvenience in everyday life, and preventive support is required for long-term care in the future. There are larger two stages. Individuals who are eligible for long-term care require nursing care immediately; this category has five stages. In both categories, the higher the number, the more severe the condition. In this study, individuals who were certified ineligible for support were at a high risk of falling into the eligible for support or eligible for long-term care categories.

The number of the subjects who had been certified as eligible for support or long-term care was as follows: those who needed support: *n* = 10; those who needed support: *n* = 3; those who needed long-term care: *n* = 7; those who needed long-term care: *n* = 14; those who needed long-term care: *n* = 4; those who needed long-term care: *n* = 4.

Based on the physicians’ diagnosis, the number of subjects with dementia or suspected of having dementia that were ineligible for support was 4, those that were eligible for support were 2, and those that were eligible for long-term care were 19.

All subjects provided their oral and written informed consent before they participated in the study. Consent was obtained from family members/parents of patients suspected of having dementia. All subjects had permission to participate in study by their primary physician. The study was conducted in accordance with the Declaration of Helsinki, and the protocol was approved by the Ethical Review Board of Tokyo Healthcare University (KYO-25-2) on April 23, 2013.

### Measurement of lower-limb muscle strength

**Fig. 1:**
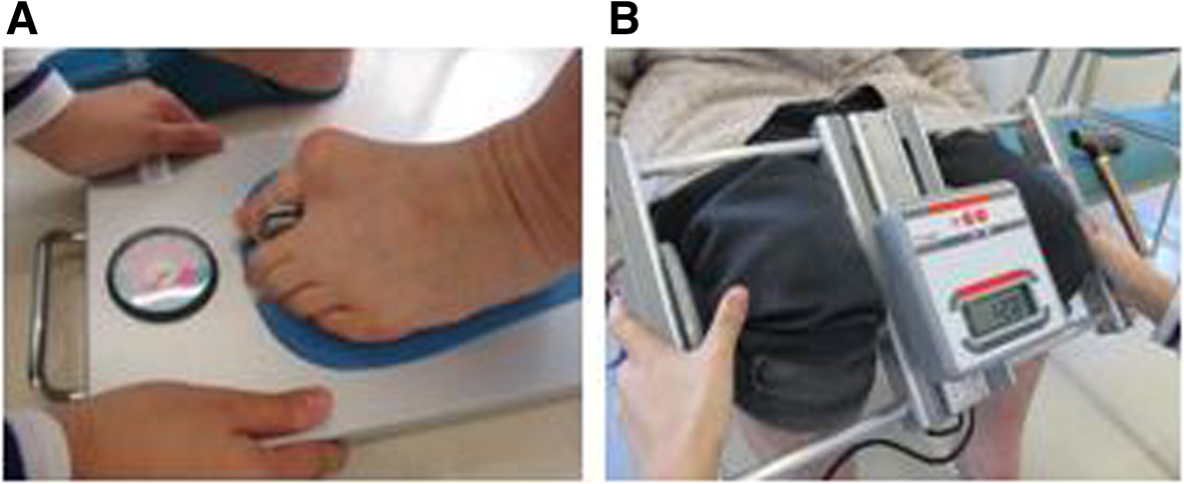
Measurement of lower-limb muscle strength. **a** Toe-gap force measurement device. **b** Knee-gap force measurement device

The participants’ physical function was measured at the senior center. Lower-limb muscle strength was assessed by measuring toe-gap (Fig. 1a) and knee-gap force (Fig. 1b) [15–18]. The device used to determine toe-gap force (Checker-Kun, Nisshin-Sangyo Inc., Tokyo, Japan) measure the clamping force between the hallux and the digitus secundus pedis and muscle strength under the knee including the anterior tibialis muscle [15]. The device used to determine knee-gap force (Hunbariryoku-Checker-Kun, Nisshin-Sangyo Inc.) measured the hip joint adductor muscle [16]. The results obtained via these devices, which showed a high correlation between the numerical values observed, and the subjects’ ability to walk 10 m were used to estimate the risk of falling in a static state [15].

Previous studies have reported that elderly people with a high risk of falling showed a toe-gap force of 2.4 kgf or lower and knee-gap force of 10 kgf or lower, at the threshold of the fall risk screening index [16, 17]. Therefore, we focused on the risk of falling in terms of lower-limb muscle strength.

### Measurement of foot pressure distribution.

**Fig. 2:**
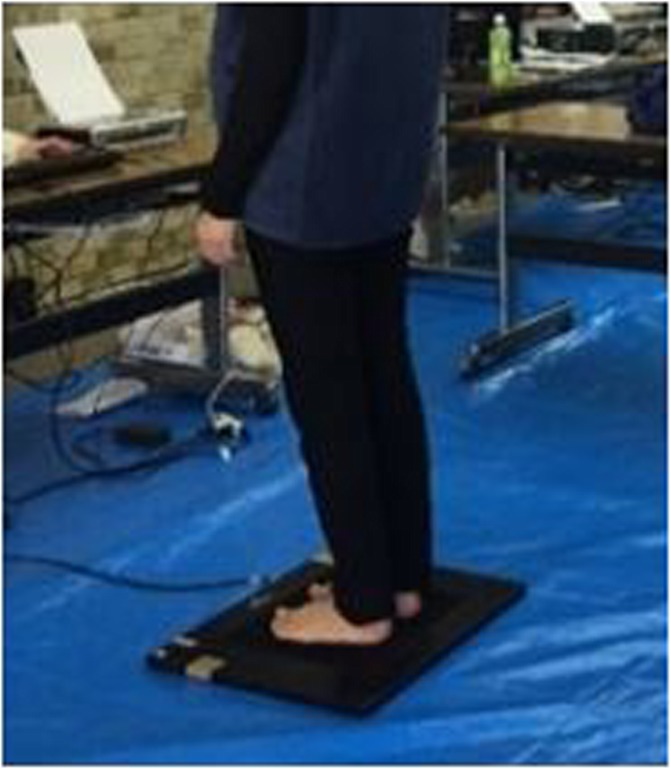
Measurement of foot pressure distribution

The load on the sole of the foot is closely related to balance, which is related to risk of falling. Therefore, the loads on the soles of the feet were measured using the twin99 (Midi Captures, Balma, France) with subjects in a static standing position, as shown in Fig. 2. Subjects were instructed to maintain a natural standing posture and the measurement process lasted 10 s. After measuring the load on the sole of the foot, and based on the distribution of weight across the entire sole, we were able to estimate the status of the musculoskeletal system, including the shapes of the toes, heel, and foot arches.

Elderly adults often raise their toes while standing still [19]; therefore, the surface of the supporting base is decreased, which reduces their balancing ability. The toes are the main determinants of postural stability and control. In addition, a study research reported that calluses form on areas of the sole on which strong pressure is; therefore, a high proportion of elderly adults who had experienced falls had developed calluses [20].

Based on the above-mentioned information, we evaluated the following factors in five areas of the foot to determine the subjects’ foot types: (1) toe-ground contact, (2) load on the forefoot, (3) load on the middle portion of the foot, (4) external load, and (5) the shape of the arches of the foot. Specifically, the effect of raised toes on balancing ability was evaluated based on the loads on the forefoot and middle portion of the foot and external load, and the muscle function and skeletal collapse on the sole of the foot according under high load was estimated. In other words, the status of the inner longitudinal arch was estimated based on the load on the forefoot and the middle portion of the foot, the status of the outer longitudinal arch was estimated based on external load, and the flexibility of the muscles of the longitudinal arch of the inner foot was estimated based on the shape of the inner longitudinal arch.

Foot problems assessed via the five items described above were categorized into one of three levels ranging from 0 to 2 (i.e., level 0, severe; level 1, mild; level 2, normal). The total scores obtained for the left and right feet were regarded as indicative of the loads on the soles. Higher total scores indicated better distribution of loads and a score of 20 for both feet was considered perfect. Improvement in foot shape was defined as an increased of more than three points over baseline after receiving foot care. Subjects in both groups whose total scores increased or decreased by two points considered to have maintained their foot shapes. Those with a decrease of more than two points were considered to have experienced deterioration of their foot shapes.

### Questionnaires

The questionnaires were administered before and after the foot care intervention to assess the subjects’ basic characteristics and backgrounds. The baseline questionnaire included items pertaining to the presence or absence of underlying diseases, the status of the feet and toenails, and previous falls; the post-intervention questionnaire examined the changes in the feet and toenails resulting from foot care.

The questionnaires also measured changes in subjects’ everyday lives, including the frequency with which they went out and interacted with friends, their feelings and behaviors, and the frequency with which they communicated with others. In addition, the questionnaires measured changes in subjects’ facial expressions, how they felt about the amount and nature of the help they received, and their activity levels.

Some of the subjects had been diagnosed with dementia and required assistance from others. Therefore, staff members at residential facilities and subjects’ family members completed a questionnaire regarding changes in subjects’ activity levels and the extent of the foot-care assistance they required following the intervention.

### Foot care intervention

Foot care technicians at the Medical Foot-Care JF Association provided foot and toenail care. The technicians were nurses with expertise and skill in foot and toenail care, including the provision of care for elderly adults and people with toe problems.

The frequency and duration of the intervention (once per month for 5 months) were determined based on the toenail growth rate and environmental changes affecting the foot. The intervention consisted of guidance on routine foot and toenail care and treatment for damaged toenails, thickening toenails, and calluses. In this study, the measurement results were given to the patients immediately. The feedback paper listed the method of care for each result, and guidance was given. Measurement of lower-limb muscle strength and plantar pressure distribution of the intervention and control groups was performed before the first foot-care treatment and 5 months later. We confirmed improvements in the appearance of the subjects’ toenails through visual inspection.

### Statistical analysis

Data analysis was performed using the Statistical Package for the Social Sciences (version 24; IBM Corp., Armonk, NY) statistical analysis software. The differences in age between the intervention group and the control group were examined using Student’s t test. In addition, the differences in age between the three intervention subgroups and the control group were examined using one-way analysis of variance. A t-test was used to examine the differences in toe-gap force, knee-gap force, and the distribution of the foot pressure between the intervention and control groups.

## Results

The control group in this study was propensity score-matched with the ineligible for support subgroup of the intervention group. Therefore, there were no age differences between. The mean ages of the control group and the certified eligible for support and long-term care subgroups differed significantly.

### Lower-limb muscle strength

**Fig. 3:**
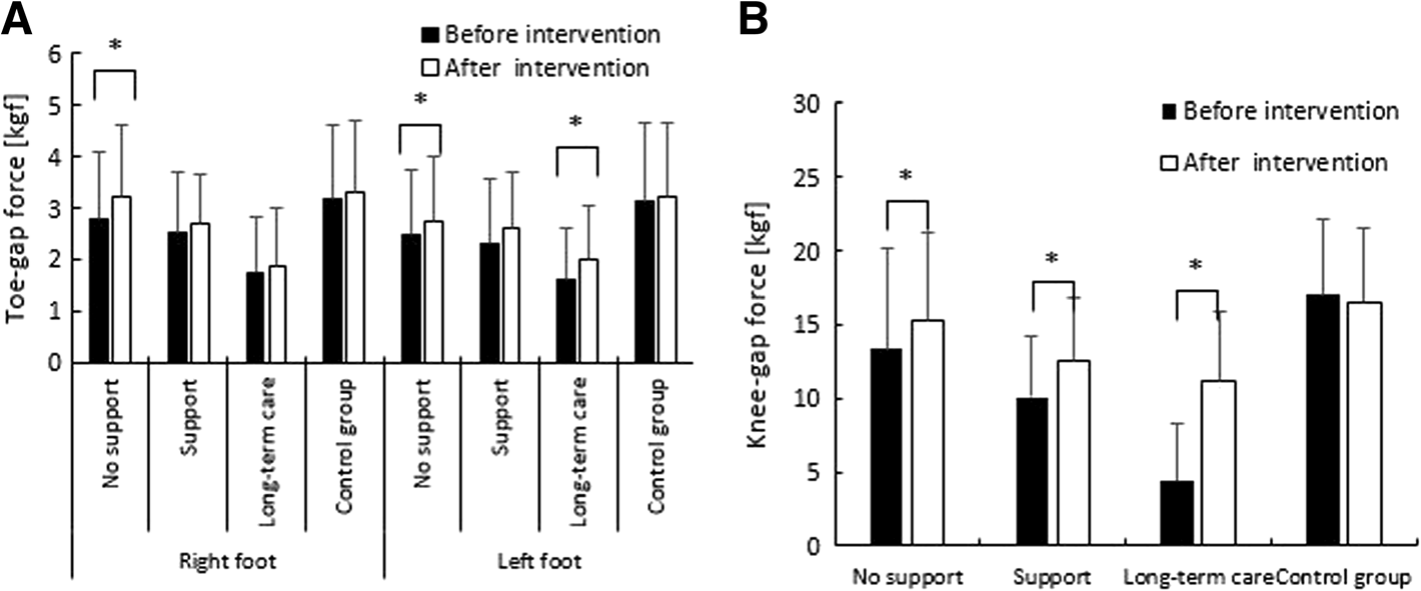
Lower-limb muscle strength findings. **a** Change in toe-gap force. **b** Change in knee-gap force

Figure 3 shows the changes in lower-limb muscle strength following 5 months of foot care. Toe-gap force in both feet improved significantly in the intervention groups (right: *p* = 0.008, d = 0.22; left: *p* = 0.003, d = 0.27). Furthermore, both the right and left feet improved by 1.0 time in the control group (right: *p* = 0.25, d = 0.09; left: *p* = 0.32, d = 0.07).

In the intervention group’s certified eligible for long-term care subgroup, 12 subjects were unable to move their toes before the intervention, as their toe-gap force was 1 kgf or lower. Of these, 9 (75%) showed improvement, and 1 was reclassified from the falling risk group to the non-falling risk group after the intervention.

With respect to the risk of falling in terms of lower-limb muscle strength, 19 subjects (59%) who were at risk of falling before the intervention, 9 (69%) who were eligible for support, and 23 (79%) who were eligible for long-term care showed improvement after the intervention. In addition, 3 (16%) subjects who were ineligible for support, 5 (56%) who were eligible for support, and 3 (13%) who were eligible for long-term care were reclassified from the falling risk group to the non-falling risk group after the intervention.

The knee-gap force improved significantly by 1.1 times in the subjects who were ineligible for support (*p* = 0.01, d = 0.31), by 1.2 times in those who were eligible for support (*p* = 0.03, d = 0.58), and by 2.6 times in those who were eligible for long-term care (*p* = 0.000, d = 1.60). In addition, knee-gap force decreased by 1.0 times in the subjects in the control group (*p* = 0.11, d = 0.12).

In the intervention group’s certified eligible for long-term care subgroup, 7 subjects whose standing posture was classified as unstable and risk of falling was considered high before the intervention due to a knee gap force ≤5 kgf, showed improvement in their force measurements after the intervention. In addition, 15 (47%), 7 (54%), and 27 (93%) subjects in the certified ineligible for support, eligible for support, and eligible for long-term care subgroups, respectively, were considered at high risk of falling before the intervention and showed improvement post-intervention.

### Foot pressure distribution

The total scores for the left and right feet improved significantly in 1.1 times, 1.1 times, and 1.1 times of the subjects in the certified ineligible for support (*p* = 0.000, d = 0.85), eligible for support (*p* = 0.004, d = 0.90), and eligible for long-term care (*p* = 0.01, d = 0.62) subgroups, respectively. In addition, the subjects in the control group showed an improvement of 1.0 times (*p* = 0.56, d = 0.07). In particular, the scores of the contact between the toes and the ground improved significantly in the intervention subgroups. The number of the subjects whose toes did not come into contact with the ground at all and whose foot problems were classified as severe (level 0) before the intervention were 16 (50%), 5 (38%), and 10 (34%) in the certified ineligible for support, eligible for support, and eligible for long-term care subgroups, respectively. Of these, 5 (31%), 4 (80%), and 5 (50%) in the certified ineligible for support, eligible for support, and eligible for long-term care subgroups, respectively, were considered to have normal toe contact with the ground after the intervention.

**Fig. 4:**
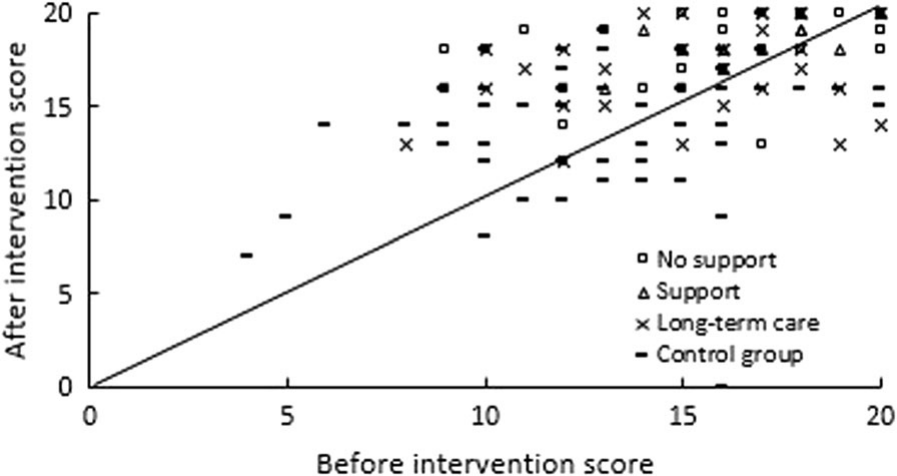
Change in foot pressure distribution scores. The scores recorded before the intervention are plotted on the horizontal axis, and those recorded after the intervention are plotted on the vertical axis. The diagonal line represents subjects who showed improvements in their foot pressure following the receipt of foot care

Figure 4 shows the differences in total foot pressure evaluation scores for the left and right feet between the three intervention subgroups and the control group. In the intervention group, 38, 57, and 5% of the subjects showed improvement, the potential for maintenance, and deterioration of their foot shapes, respectively. In the control group, 33, 48, and 19% showed these respective changes.

In the certified ineligible for support subgroup, 38, 59, and 3% of the subjects showed improvement, the potential for maintenance, and deterioration of their foot shapes, respectively. In the certified eligible for support subgroup, 31, 69, and 0% of the subjects showed these respective changes. In the certified eligible for long-term care subgroup, 41, 55, and 5% of the subjects showed these respective changes.

**Fig. 5:**
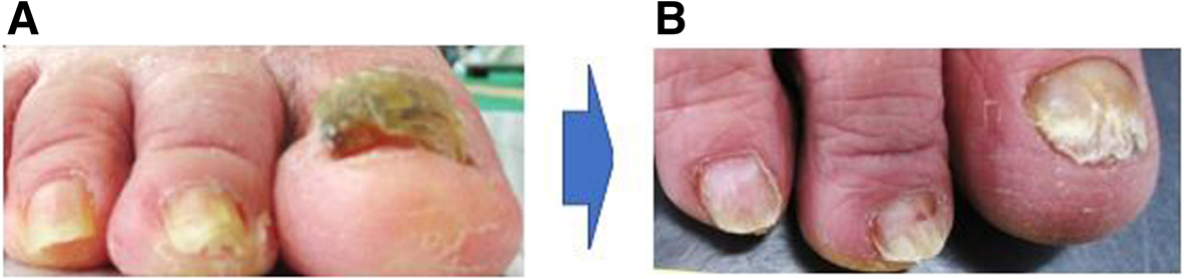
Change in the toenails of a subject in the intervention group. **a** Before the intervention. **b** After the intervention

Figure 5 shows the changes in the toenails of one of the subjects in the intervention group. The toenails had thickened considerably, but 5 months after the intervention, the thickening had been reduced to the extent that the nail appeared normal.

### Questionnaire results

With respect to falls before the intervention, 30% of the subjects in the intervention group had experienced a fall during the preceding year. In addition, 85% of the subjects in the intervention group reported abnormalities, such as hallux valgus, curved toenails, and pain caused by calluses, in their feet and toenails. Furthermore 89, 92, and 79% of the subjects in the certified ineligible for support, eligible for support, and eligible for long-term care subgroups, respectively, reported abnormalities in their feet. The results showed that 88, 91, and 50% of the subjects in the certified ineligible for support, eligible for support, and eligible for long-term care subgroups, respectively, reported improvements in their feet and toenails following the receipt of foot care.

The subjects reported that, since receiving foot care, their ability to put their socks or stockings on without assistance had returned, because their toenails no longer became caught. They were able to walk without experiencing anxiety because of a reduction in the pain caused by their curved toenails.

The assessment of changes in the daily lives of the subjects in the intervention groups, via the questionnaire items pertaining to how they felt each day, showed that 64, 58, and 57% of the subjects in the certified ineligible for support, eligible for support, and eligible for long-term care subgroups, respectively, reported that they had become happier and more cheerful and had a more positive outlook on life since receiving foot care. In addition, 40, 25, and 26% of the subjects in the certified ineligible for support, eligible for support, and eligible for long-term care subgroups, respectively, reported that they had begun to go out more frequently since receiving foot care. Moreover, 47, 50, and 61% of the subjects in the certified ineligible for support, eligible for support, and eligible for long-term care subgroups, respectively, reported that the range of their activities had expanded. Specifically, the subjects’ questionnaire responses indicated that they had changed their habits in their daily lives since receiving foot care; for example, they had begun to take trips, walk their dogs, and go shopping. Furthermore, 38, 27, and 45% of the subjects in the certified ineligible for support, eligible for support, and eligible for long-term care subgroups, respectively, reported that they had begun to engage in social exchanges with friends more frequently since receiving foot care.

The results of the questionnaire completed by staff members at residential facilities and family members showed that 62, 75, and 41% of the subjects in the certified ineligible for support, eligible for support, and eligible for long-term care subgroups, respectively, had increased their activity levels since receiving foot care. Furthermore, 67, 25, and 50% of the subjects in the certified ineligible for support, eligible for support, and eligible for long-term care subgroups, respectively, had begun to display cheerful facial expressions and smile more often because of their increased activity levels. Moreover, they had begun to show appreciation for the assistance provided by staff members at residential facilities and their family members. In addition, 10, 25, and 31% of the subjects in the certified ineligible for support, eligible for support, and eligible for long-term care subgroups required less assistance since receiving foot care.

In addition, some of the subjects reported looking forward to the pleasure of receiving foot care each month during the 5 months of the intervention and engaged in voluntary work that involved informing their friends about the importance of foot and toenail care.

## Discussion

In our study, 85% of participants reported abnormalities, such as hallux valgus, curved toe nails, and pain caused by calluses, in their feet and toenails before receiving foot care, which was similar to reported rates in previous studies [4, 5]. In addition, their risk of falling was considered high, as many of the subjects had experienced falls in the past and showed toe- and knee-gap forces below the fall risk threshold.

As shown in Fig. 3, toe-gap force in both feet improved in all three intervention subgroups; however, only maintenance, rather than improvement, of the toe-gap force was observed in the control group. The rate of improvement in the intervention group was considerable, particularly in subjects whose physical function levels were so low that they had been certified eligible for long-term care and those whose toe gap force was 1 kgf or lower before the intervention. During the measurement of toe-gap force, subjects who experienced forefoot pain exhibited a lack of strength in their feet. Therefore, the pain relief resulting from foot care is likely to have restored the functionality of their toes, increasing movement and toe-gap force.

The results demonstrated that regular foot and toenail care improved toe strength, even in frail elderly adults. The toes contribute to posture control and the ability to walk; therefore, improvements in toe strength and movement, and subsequent reduction in the risk of falling, likely lead to the improvement and maintenance of the ability to perform ADL effectively.

The knee-gap force assessments (Fig. 3) showed significant improvements in all three intervention subgroups. Seven subjects in the certified eligible for long-term care subgroup that had a force of 5 kgf or lower and were at high risk of falling before the intervention showed considerable improvement; in addition, some subjects improved to the extent that they were reclassified from the falling risk group to the non-falling risk group. The questionnaires completed by the subjects, their family members, and staff members at residential facilities indicated that the reasons for these improvements included the benefits of foot care and increased function in their feet and toenails, which motivated them to become interested in caring for their feet. Considering that the subjects had made an effort to stand and to walk, walking is likely to have strengthened the inner hip joint muscles and led to improvement in movement.

As shown in Fig. 4, we performed a quantitative evaluation of the status of the loads on the soles of the feet, which is associated with balance. Total scores increased significantly in all three intervention subgroups but decreased in the control group. In the intervention subgroups, some subjects’ toes had improved to the extent that they were able to make full contact with the ground, which secured the surface of the supporting base and increased the likelihood of assuming a stable standing position. This was attributed to increased strength in the toes, as the subjects had experienced difficulty putting weight on their toes because of forefoot pain. Relief of that pain via foot care allowed them to put weight on their toes.

With respect to the factors related to increased scores for contact between the toes and the ground, which reflected foot pressure, the toenails support toe strength, allowing the toes to touch the ground. When the toenails are thickened, as shown in Fig. 5, it is difficult for the toes to make full contact with the ground. Observation of the movement of the toes suggested that restoring the toenails to their normal state via foot care resulted in increased toe strength, which allowed the subjects to exert pressure on the soles of their feet.

Furthermore, foot care led to positive changes in the subjects’ behaviors and feelings. This was attributed to improvements in physical function, which allowed subjects to stand firmly on the ground and walk steadily. As they were able to walk without experiencing anxiety regarding falling, they regained their motivation for walking, which extended the range of their activities to include pursuits such as shopping and travelling. Overall, the ability to walk increased the subjects’ levels of motivation and led to changes in their daily lives. Moreover, we observed changes in the subjects’ attitudes, including their desire to actively engage in social activities such as voluntary work.

The above-mentioned results indicated that foot care improved physical function and reduced the risk of falling because it improved lower-limb muscle strength and foot pressure, even in frail elderly adults. Moreover, the results demonstrated improvements in the subjects’ psychological health and ability to perform ADL; therefore, foot care is important in the prevention of frailty and long-term supportive care.

As the subjects in the control group were younger relative to those in the intervention group, the proportion of those at risk of falling was lower. Therefore, we expected their toe- and knee-gap forces to be maintained; however, the assessment indicated that it decreased. Furthermore, foot pressure distribution was likely to decrease in the control group. Therefore, we conclude that elderly adults whose physical and foot functions continue to deteriorate should receive foot care and be certified eligible for support and long-term care to ensure that they will be able to maintain their function if their frailty increases.

A previous study reported that deformation of the forefoot, including hallux valgus, influences quality of life in elderly adults [21]. When hallux valgus occurs, calluses are more likely to form in the forefoot, which affects gait. In addition to hallux valgus, in the case of diabetes with chronic diseases often found in middle-aged and elderly adults, the curved toenails and the foot of the sole become risk factors for lower-limb amputation.

An incorrect shoe size can have an effect on falls and quality of life [10, 22, 23]. In the context of toenail thickening and curvature, pain may occur when wearing shoes of the correct foot size; therefore, these elderly adults may be wearing shoes that are too big for them. Thus, it is important to provide education on correct shoe sizing in addition to foot care to prevent falls and improve quality of life.

### Limitations

This study has certain limitations in terms of subject selection and sample size. In this study, foot care technicians provided foot and toenail care to the subjects. The number of foot care technicians was limited, and the number of subjects was small. Since a larger number of subjects were needed for a robust analysis, we employed a comparatively larger, propensity-score matched control group.

The factors considered when assigning support and long-term care eligibility included dementia. Therefore, this study selected subjects with dementia but did not assess cognitive functions using tests such as the mini-mental state examination. Dementia was diagnosed based on the physicians’ diagnosis, and we did not examine changes in cognitive function after foot care. Based on the questionnaire results of the intervention group, including individuals with dementia, the subjects’ daily mood improved, and they went out more frequently than before foot care. Since elderly adults experience a decreased engagement in conversation and activities, an increase is noteworthy.

## Conclusions

This study involved the provision of foot care to frail elderly adults for 5 months and assessed its contribution to fall prevention, quantitative evaluation of lower-limb muscle strength and foot load, which is prospective of physical function. Furthermore, it examined changes in subjects’ feelings and behaviors resulting from the receipt of foot care. The alleviation of the subjects’ foot and toenail problems via sustained foot care improved the physical function, including lower-limb muscle strength and foot load, even in frail elderly adults. This indicated that it contributed to a reduction in the risk of falling as assessed by lower-limb muscle strength, increased activity levels, and resulted in more positive attitudes as subjects’ psychological health improved.

## Abbreviations

ADL: Activities of daily living
